# Relationship between students’ perceptions of the adequacy of M1 and M2 curricula and their performance on USMLE step 1 examination

**DOI:** 10.1186/s12909-019-1796-3

**Published:** 2019-09-14

**Authors:** Mohammed K. Khalil, William S. Wright, Kelsey A. Spearman, Amber C. Gaspard

**Affiliations:** University of South Carolina School of Medicine Greenville, South Carolina, 607 Grove Road, Greenville, SC 29605 USA

**Keywords:** USMLE step 1, Curriculum evaluation, Undergraduate medical education

## Abstract

**Background:**

Performance on United States Medical Licensing Exam® (USMLE®) Step 1 examination (Step 1) is an important milestone for medical students. It is necessary for their graduation, and selection to interview for the National Resident Match Program®. Success on Step 1 examination requires content alignment, and continuous evaluation and improvement of preclinical curriculum. The purpose of this research was to observe the association between students’ perceptions of deficits in the curriculum based on core disciplines and organ systems in relation to students’ performance in those disciplines and systems on USMLE® Step 1 examination.

**Methods:**

An anonymous survey with closed-ended and open-ended questions was sent to 174 medical students, the class of 2018 (77), and 2019 (97) within 2–3 weeks of taking Step 1 examination. Students’ feedback as well as students’ performance on Step 1 examination were organized into disciplines and organ systems to allow for more specific curriculum analyses. The closed-ended questions provide three selections (yes, no and not sure) regarding students’ agreement to the adequacy of M1 and M2 curricula to prepare students for Step 1 examination. Students’ responses on the closed-ended questions were reviewed in conjunction with their Step 1 performance. The open-ended feedback was qualitatively analyzed for emergent themes or similarity with closed-ended questions in identifying any shortcoming of the curriculum.

**Results:**

The data show an apparent relationship between students’ evaluations and students’ performance on Step 1 examinations. A high percentage of students’ disagreement of the curriculum adequacy was also reflected in a lower performance on Step 1 examination. Additionally, the themes that emerged from the qualitative analysis have confirmed the areas of curricular deficiency.

**Conclusion:**

The data collected from this research provides insight into the degree of usefulness of students’ evaluations as a way of assessing curriculum deficits in preparing students for their Step 1 examination.

## Background

Although different comprehensive evaluation models [1–3] are available for curriculum evaluation, medical schools are constantly looking for innovative ways to evaluate their curricula, especially if the evaluation method responds efficiently in making recommendations and changes. For instance, Chang et al. [4] utilized medical student graduates’ ability to match in highly regarded residency programs as a measure of undergraduate medical education program quality. They also reported that key factors in students finding “elite” residency programs were “clerkship grades and Step 1 scores” [4]. This finding concerning United States Medical Licensing Exam® (USMLE®) Step 1 (“Step 1″) scores is consistent with the fact that Step 1 score is a top factor used for the National Resident Match Program® to select applicants to interview [5], despite calls to reevaluate the role of Step 1 in residency selection [6]. To this end, this study aims to evaluate the degree of usefulness of students’ evaluation as a way of efficiently assessing curriculum deficits in preparing students for taking Step 1 examination.

Medical education has evolved since the revolutionary report on medical education by Flexner in 1910 [7] with new recommendations for medical education recently written [8]. In addition, to these seminal documents, schools have been trying to improve medical education curricula to integrate cultural competence [9], palliative education [10], population health [11], and use collaborative approaches to evaluate and transform medical curriculum [12]. While medical school curricula are evolving to address changing needs, students must complete Step 1 which measures basic science knowledge [13]. Due to the stated importance of Step 1 examination and content covered, a primary goal for medical schools within the first two years of the curriculum is preparing students for Step 1 examination. With that said, multiple data points are important in the development of curriculum as expressed in the six-step approach for curriculum development [14]. The six-step approach for curriculum development has been used to address specific aspects of the curriculum [15–17]. Denney-Koelsch et al. [17] surveyed clerkship and course directors, and used students’ evaluations of courses to determine if topic areas are covered in the curriculum. Day et al. [18] reported curricular changes to improve musculoskeletal curriculum following student feedback, while Baroffio and Gerbase [19] reported students’ perceptions being used for changes in problem-based learning. However, detailed reports of curricular revision specific to global (i.e. all content areas) Step 1 performance is limited.

One direct measure of global performance on Step 1 is provided in March of the year following student examination (i.e. March of 2018 for students taking Step 1 in calendar year 2017). Although relatively timely, this can be up to nine months following examination by a cohort of students and curricular development for the upcoming academic year has typically been completed by this time for many schools. In addition, the information provided is limited. Currently, the annual report of student performance on Step 1 includes a performance summary, score histogram, and a score plot. The score plot divides the performance of examinees taking Step 1 into three content areas: 1) physician task, 2) discipline, and 3) system. The score plot for an individual school provides the mean performance for test takers in each of the content areas, and one standard deviation from the schools’ mean compared to the national mean in each content area. Although the content areas are provided in the feedback from the USMLE®, the association between data reported by the USMLE® and students’ perceptions of preparedness is lacking. As such, feedback from individual learners is an important measure that should be considered during program level evaluations [14]. Therefore, the aim of this study was to 1) determine students’ perceptions of preparedness for Step 1 examination, and 2) determine if there is association between student’s perceptions of preparedness for the Step 1 examination in various disciplines and organs systems and their performance reported on the score plot provided to schools for Step 1 examination.

## Methods

### Participants

Second-year (M2) students from the classes of 2018 (*n* = 77), and 2019 (*n* = 97) were recruited to participate in the study within 2–3 weeks of their first attempt in taking Step 1 examination. Ninety-nine students responded to the anonymous survey for a response rate of 57% for both classes. Participants were 55% females and 45% males. Their ages ranged from 21 to 34 years with an average age of 23 years. Participants’ average UGPA was 3.65 on a 4-point scale, and their overall MCAT percentile rank was 70%.

### Data collection

An anonymous survey with closed-ended and open-ended questions was sent to 174 medical students within 2–3 weeks of taking Step 1 examination. Two closed-ended questions assessed students’ agreement to the adequacy of M1 and M2 curricula to prepare students for Step 1 examination. Students selected between yes, no and not sure for each question to report if they feel the curriculum sufficiently covers disciplines and organ systems within the M1 and M2 curriculum. At the end of the survey, students were also asked to identify the specific content areas that were not sufficiently covered by M1 and M2 curricula. Performance of students in disciplines and organ systems on the Score Plot reports provided by the National Board of Medical Examiners® taking the Step 1 for first-time takers were used to identify the least performing disciplines and organ systems.

### Data analysis

Students’ feedback as well as first-time test taker performance on Step 1 examination were organized into curricular disciplines and organ systems to allow for more specific analyses of the curriculum. Students’ responses on the survey were analyzed to identify the top curricular disciplines and organ systems identified by students as insufficiently covered by the curriculum. Thereafter, these identified shortcomings were compared to the least performing disciplines and organ systems on Step 1 examination (i.e., disciplines and organ systems performing at or less than − 0.2 standard deviation (SD) on the score plot of our medical school relative to the distribution for all US/Canadian schools). The open-ended feedback was qualitatively analyzed to identify emergent themes or patterns of students’ perceptions. A search was conducted for meaningful word repetition (e.g., pharmacology for discipline; cardiovascular for organ system) across all student responses. The number of agreements in identifying insufficient disciplines and organ systems were compiled and presented as percentages to indicate word frequencies in relation to the total responses. The open-ended feedback was reviewed with closed-end feedback and student performance for triangulation of data.

## Results

**Fig. 1:**
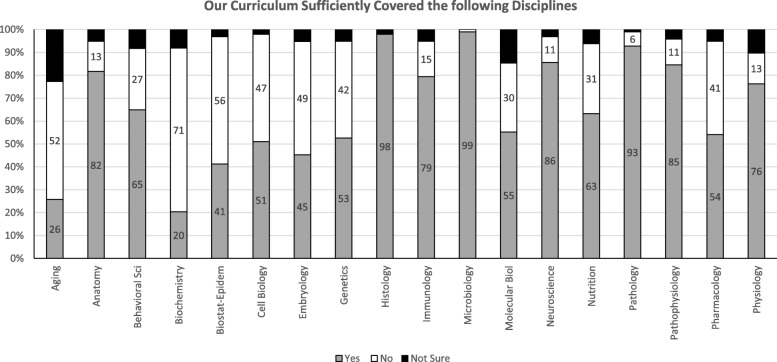
Students’ perceptions of the adequacy of the curriculum to sufficiently cover curricular disciplines using closed-ended questions (i.e. “yes, no, not sure” scale). Legend: Percent of students responding yes, no, or not sure to closed-ended questions on disciplines covered within the first two years of the undergraduate medical education curriculum. *N* = 99 students, Behavioral Sci = Behavioral Sciences, Biostat-Epidem = Biostatistics and Epidemiology

**Fig. 2:**
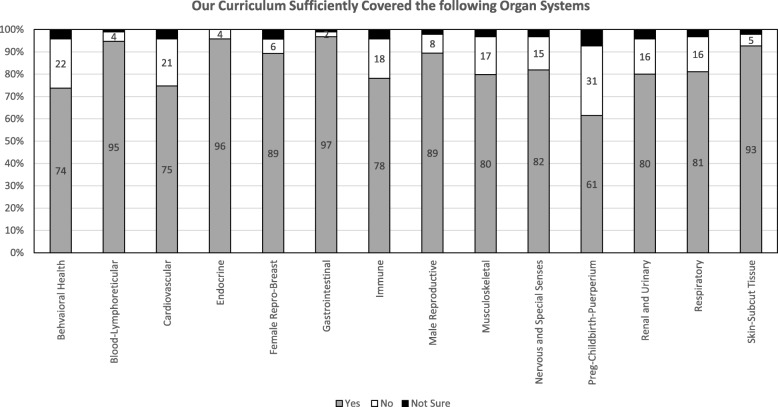
Students’ perceptions of the adequacy of the curriculum to sufficiently cover organ systems using closed-ended questions (i.e. “yes, no, not sure” scale). Legend: Percent of students responding yes, no, or not sure to closed-ended questions on organ systems covered within the first two years of the undergraduate medical education curriculum. N = 99 students, Preg-Childbirth-Puerperium = Pregnancy-Childbirth-Puerperium

Based on the 99 student responses, the top disciplines identified as not adequately covered by the curriculum are: biochemistry (71%), biostatistics and epidemiology (56%), aging (52%), embryology (49%), cell biology (47%), genetics (42%), and pharmacology (41%) (Fig. 1). The top organ systems identified as insufficiently covered by the curriculum are: pregnancy, childbirth and puerperium (31%), behavioral health (22%), cardiovascular (21%), and immune (18%) (Fig. 2).

Areas identified as insufficiently covered by M1 and M2 curricula based on students’ responses to an open-ended question are: biochemistry (59%), anatomy-embryology (35%), pharmacology (32%), behavioral sciences (28%), and physiology (21%). This qualitative analysis with examples of student’s responses is summarized in Table 1.

**Table 1:** Qualitative analyses of students’ shared perceptions regarding insufficient disciplines and organ systems

Content insufficiently covered	Example of student’s response
Biochemistry (46/78 = 59%)	“clinically relevant biochemistry, such as specific clinical presentations of particular metabolic diseases” “Biochemistry (so many things were brushed over in biochemistry. 1 little worksheet that had cystic fibrosis as an answer choice does not count as teaching CF.and that happened with many of the major biochemical diseases” “In biochemistry, the enzyme deficiency diseases need to be covered more thoroughly.”
Anatomy and Embryology (27/78 = 35%)	“I feel that embryology needs to be covered in more detail in both the first and second year. I feel that this was the main way that anatomy was tested for me for Step 1 and I think I would have benefitted from a heavier dose of this material in our curriculum.” “I feel that there was not enough coverage during M2 for embryology. Even a 30 min “intro” to start each module would have gone a long way to help us retain this information.”
Pharmacology (25/78 = 32%)	“Pharm: top 5 tested/most pertinent drugs should be taught during M1 for a foundation for M2...the remainder added in M2 and most important reiterated.” “Pharm was not sufficiently covered during the M2 curriculum. There were countless drugs taught that were not tested on step 1 and countless drugs on step 1 that were not taught. The information taught about each drug was not relevant in most cases and pharm vignettes were not representative of step 1 material.”
Behavioral Sciences (22/78 = 28%)	“Behavioral science and biostatistics were covered so last minute in the M2 curriculum that I didn’t feel that we were given enough time to absorb and fully understand the important concepts.” “Biostats and epidemiology curriculum is severely lacking in our M1 and M2 curriculum.”
Physiology (16/78 = 21%)	“Physiology - this topic is very broad, but for me I found that it was a weak point going into studying for STEP 1. Most of physiology was covered in the M1 year and I know the topics were covered but not very well. Cardiovascular physiology and renal physiology were the two areas that I struggled with the most M1 year and during Step 1 study.” “Cardiovascular was not sufficiently covered. We spent way too much time interpreting EKGs and not enough time on basic physiology and pathophysiology.”

On the score plot, Step 1 score distribution of our medical school relative to the distribution for all US/Canadian schools showed that behavioral sciences, biochemistry, genetics, gross anatomy and embryology, histology and cell biology, nutrition, pathology, pharmacology, and physiology are the lowest performing disciplines at − 0.2 SD or lower. Immune, behavioral health & nervous systems/special senses, cardiovascular, respiratory, gastrointestinal, and endocrine systems are the lowest performing organ systems at − 0.2 SD or lower.

Comparing the content areas of insufficiency identified by students’ responses to the (yes, no, not sure) scale, and their responses to the open-ended question showed comparable results (Table 2). Interestingly, areas of insufficiencies coincide with the least performing disciplines and organ systems on Step 1 examination (Table 2).

**Table 2:** Comparison of USLME Step 1 disciplines scoring ≤ − 0.20 standard deviations (SD) below the national mean and areas of curricular insufficiency identified by students using the closed-ended scale “yes, no, not sure”-, and their response to the open-ended question to students’ performance on Step 1 examination

USMLE Step 1 Discipline (Students’ Performance (− 0.20 SD)	Insufficiency of Disciplines Identified by closed-ended questions (percent of Students Responding “No” to closed-ended questions)	Insufficiency identified by the open-ended question (percent of students identifying area as insufficient)
Behavioral Sciences (− 0.20)	Biostatistics and Epidemiology (56%)	Behavioral sciences (28%)
Biochemistry (− 0.30)	Biochemistry (71%)	Biochemistry (59%)
Genetics (− 0.38)	Genetics (42%)	
Gross Anatomy and Embryology (−0.43)	Embryology (49%)	Gross Anatomy and Embryology (35%)
Histology and Cell Biology (−0.20)	Molecular Biology (30%)/Cell Biology (47%)	
Pathology (−0.20)		
Pharmacology (−0.23)	Pharmacology (41%)	Pharmacology (32%)
Physiology (−0.25)		Physiology (21%)

## Discussion

In the present study, we evaluated the adequacy of our curriculum to cover the contents tested in the USMLE® Step 1 examination from the medical students’ perspectives, and we assessed if inadequacy is associated with students’ performance. After taking the United States Medical Licensing Exam® (USMLE®) Step 1 examination, medical students identified the disciplines and organ systems that they perceived were insufficiently covered by our curriculum. These identified inadequate contents were also shown to be the low performing disciplines and organ systems on Step1 results.

USMLE® Step 1 examination is a major milestone for the progression of medical students through the medical school curriculum, and their success to be selected for an interview and to match for residencies [5]. Many medical schools have tried to develop evaluation models to predict students’ success on this major examination [20–22]. Most of these models relied on pre-matriculation and internal students’ academic performance data, as well as students’ behavior and acquisition of learning strategies skills [23]. However, faculty effectiveness, learning environments and medical school curricula can also be contributing factors that affect students’ performance on Step 1 examination. In addition, these models do not address methods used to evaluate the medical education curriculum. Our findings suggest student performance should be one of several metrics used for curricular revision.

Curricular insufficiencies identified by students in the closed-ended questions are consistent with those insufficiencies identified in the open-ended question for the disciplines within our curriculum. Biostatistics-epidemiology, biochemistry, embryology, genetics, and pharmacology were seen as the major disciplines that were not sufficiently covered by our curriculum. These insufficiencies were reflected in lower scoring disciplines reported on Step 1 score plot provided by the USMLE®. An interesting finding is that students’ perceptions of insufficiency in our program corresponded with Step 1 students’ performance in the disciplines reported on Step 1 score plot. Despite the notion that medical students heavily utilize external study resources while preparing for Step 1 examination [24], our study suggests that perceived inadequacies within the medical school curriculum may be related to student performance. In addition, it was previously reported that in our curriculum, the overall weighted basic science performance explains 43.56% (M1), 51.41% (M2), and 57.31% (M1 and M2) of Step 1 score variations [23]. This confirms the importance of the basic science curriculum in preparing students for Step 1 examination. Indeed, a national basic science curriculum has been proposed for schools to address Step 1 necessary contents [25].

The magnitude of students’ perceptions of the organ systems insufficiencies were not as obvious as the disciplines insufficiencies. However, with the exception of pregnancy, child birth and puerperium (31%), the top listed insufficiently covered organ systems identified by closed-ended question: behavioral health (22%), cardiovascular (21%), and immune (18%) coincide with students’ low performance on Step 1 in the disciplines reported on Step 1 score plot. It is possible that the difference in magnitude between students’ perception of insufficiency in disciplines versus systems may be due to the design of our curriculum. Currently, two modules in the first year (totaling half of the academic year) and all modules in the second year are organ-systems based. Specific disciplines such as biochemistry, genetics, gross anatomy and embryology, histology and cell biology, and physiology are taught in the first year and are embedded in the integrated modules. Pharmacology, microbiology, and pathology are disciplines taught throughout all organ-systems based modules in the second year. This layout may give the perception to students that there is more coverage of a specific organ-system and less coverage of a specific discipline. However, we also observed discrepancies between students’ perceptions of the adequacy of disciplines and their correlated performance on Step 1 examination. For example, the discipline of pathology was among those in which the students performed below the national average by at least 0.2 SD, but it was rated as sufficiently covered in the curriculum. Although there is no clear explanation for this discrepancy, the integrated nature of Step 1 questions that include many disease processes might not be recognized as pathology questions. These limitations warrant further research.

Content analyses to identify gaps in achieving the intended goals and objectives of the medical curriculum can be a tedious task. Alternatively, the collection of student perceptions regarding the adequacy of the preclinical curriculum soon after taking Step 1 examination may prove insightful. Although collection of course and module-level students’ evaluations is a routine practice, evaluation of student perception of curriculum adequacy to prepare them for Step 1 examination is a potentially valuable supplemental tool in the continuous quest to prepare medical students for their licensing examinations. As such, the analyses of this study were shared with faculty during our annual curriculum retreat. The information was perceived useful by faculty in re-examining the selection of content and topics to be delivered during their modules. We continue to collect this data to evaluate the impact of the corresponding curricular changes on students’ Step 1 examination performance. The dynamic effort of engaging faculty to reflect on students’ perceptions is an excellent model of student-faculty partnership for an on-going curricular assessment. The idea of involving students in the development and evaluation of the curriculum has been introduced in medical education [26–28], and is believed to enhance engagement, motivation and enthusiasm [26], and help in making real-time improvement [28].

The collection of students’ perceptions on the adequacy of M1 and M2 curriculum to prepare them for Step 1 examination provide a valid and timely assessment of the curriculum. Due to the fact that medical schools receive the complete analyses of students’ performance on Step 1 examination after up to nine months following examination dates, it’s difficult to address any curricular shortcomings in a timely fashion. However, receiving students’ feedback within two weeks of taking Step 1 examination is very helpful in making necessary changes to the curriculum for the upcoming academic year.

There are several limitations to this study. First, the research was conducted in one medical school using student perceptions that were self-reported. However, as we continue to collect the data annually, the year-to-year comparison of curricular deficit in response to change provides continuous quality improvement of our curriculum. Second, the study did not evaluate the statistical correlation between individual students’ performance and their perceptions due to the anonymous nature of the survey used to collect the data. Finally, student perceptions of curricula and student performance may be influenced by multiple factors (e.g. teaching methods, learning environments, and medical school curricula); however, these factors were not a focus of this survey.

## Conclusion

The data collected from this research provides insight into the degree of usefulness of students’ evaluation as a way of assessing curriculum deficits in preparing students for taking Step 1 examination. The association between students’ perception of curriculum adequacy and students’ performance indicates that students’ evaluation is a worthy means of assessing curriculum efficacy, and a valuable tool to be utilized for the improvement of curriculum in preparing students for their Step 1 examination.

## Data Availability

The datasets used and/or analyzed during the current study are available from the corresponding author on reasonable request.

## Abbreviations

Behavioral Sci: Behavioral Sciences
Biostat-Epidem: Biostatistics and Epidemiology
Preg-Childbirth-Puerperium: Pregnancy-Childbirth-Puerperium
SD: standard deviation
Step 1: Step 1 examination
USMLE®: United States Medical Licensing Exam®
